# High throughput small RNA and transcriptome sequencing reveal capacitation-related microRNAs and mRNA in boar sperm

**DOI:** 10.1186/s12864-018-5132-9

**Published:** 2018-10-11

**Authors:** Yuan Li, Rong-Hong Li, Ming-Xia Ran, Yan Zhang, Kai Liang, Ying-Nan Ren, Wen-Cheng He, Ming Zhang, Guang-Bin Zhou, Izhar Hyder Qazi, Chang-Jun Zeng

**Affiliations:** 1 College of Animal Sciences and Technology, Sichuan Agricultural University, Chengdu, 611130 Sichuan China; 2 Farm Animal Genetic Resources Exploration and Innovation Key Laboratory of Sichuan Province, Sichuan Agricultural University, Chengdu, 611130 Sichuan Province China; 3 Department of Veterinary Anatomy & Histology, Shaheed Benazir Bhutto University of Veterinary and Animal Sciences, Sakrand, Sindh 67210 Pakistan

**Keywords:** Boar, Sperm capacitation, miRNAs, Transcriptome, High throughput sequencing

## Abstract

**Background:**

Capacitation, a prerequisite for oocyte fertilization, is a complex process involving series of structural and functional changes in sperms such as membrane modifications, modulation of enzyme activities, and protein phosphorylation. In order to penetrate and fertilize an oocyte, mammalian sperms must undergo capacitation. Nevertheless, the process of sperm capacitation remains poorly understood and requires further elucidation. In the current study, via high throughput sequencing, we identified and explored the differentially expressed microRNAs (miRNAs) and mRNAs involved in boar sperm capacitation.

**Results:**

We identified a total of 5342 mRNAs and 204 miRNAs that were differentially expressed in fresh and capacitated boar sperms. From these, 12 miRNAs (8 known and 4 newly identified miRNAs) and their differentially expressed target mRNAs were found to be involved in sperm capacitation-related PI3K-Akt, MAPK, cAMP-PKA and Ca^2+^signaling pathways.

**Conclusions:**

Our study is first to provide the complete miRNA and transcriptome profiles of boar sperm. Our findings provide important insights for the understanding of the RNA profile in boar sperm and future elucidation of the underlying molecular mechanism relevant to mammalian sperm capacitation.

**Electronic supplementary material:**

The online version of this article (10.1186/s12864-018-5132-9) contains supplementary material, which is available to authorized users.

## Background

Capacitation, a prerequisite for oocyte fertilization, is a complex process involving a series of structural and functional changes in sperms such as membrane modifications, modulation of enzyme activities, and protein phosphorylation. In order to penetrate and fertilize an oocyte, mammalian sperms must undergo hours of in vivo (in female reproductive tract) or in vitro capacitation process immediately after ejaculation [1, 2]. Multiple physiological and biochemical changes are involved in sperm capacitation [3], including protein tyrosine phosphorylation [4], membrane cholesterol efflux [5], production of reactive oxygen species (ROS) [6], membrane hyper-polarization [5, 7], as well as increase in intracellular pH [8], Ca^2+^, cyclic adenosine monophosphate(cAMP) [9], superoxide anion levels [10], and HCO_3_^−^ concentration.

Furthermore, during cryopreservation, sperms are reported to experience capacitation-like changes [8] or “cryo-capacitation” [11, 12]. These changes include increased sperm capacitation, plasma membrane reorganization, ROS generation, increased intracellular Ca^2+^ and protein tyrosine phosphorylation (PTP) [13]. Although cryo-capacitated sperms exhibit striking differences in patterns of PTP compared to normal in vitro capacitated sperms [14], cryo-capacitation should not be regarded as true capacitation [15]. It has been reported that various protein kinases and protein phosphatases are present in mammalian sperm and are implicated in sperm motility acquisition, capacitation and acrosome reaction [16, 17]. For example, tyrosine phosphorylation of sperm flagellar proteins is related to acquisition of hyperactive motility [18, 19]. Protein tyrosine phosphorylation involves three pathways: cAMP-dependent protein kinase A (cAMP-PKA) signaling pathway [20], phosphatidylinositol-3-hydroxycarboxylase (PI3K) signal Pathway [21] and mitogen-activated protein kinase (MAPK) signaling pathway [22].

In addition, optimal concentration of Ca^2+^ can promote protein phosphorylation and sperm motility [23], therefore, Ca^2+^ signaling pathway is essential for the regulation of capacitation. Furthermore, many important tyrosine phosphorylated proteins are reportedly associated with capacitation, such as protein A-kinase anchoring proteins (AKAPs) in human spermatozoa [24, 25], A-kinase anchoring protein 4 (AKAP4) in hamster sperm [26], proacrosin binding protein/p32 in boar [17, 27] and calcium-binding and tyrosine phosphorylation-regulated protein (CABYR) in mouse sperm [28].

Sperms are highly differentiated and specialized cells, their main function is to transmit paternal genetic information and coding, noncoding RNAs to the oocyte [29]. Sperm contains an array of RNAs, including messenger RNAs (mRNAs), ribosomal RNAs (rRNAs) and small RNAs [30], which are residues from the process of spermatogenesis [31–33]. Sperm RNAs may contribute to sperm movement, capacitation, fertilization and early embryogenesis [34].

In 2006, Gur and Breitbart have demonstrated that labeled amino acids are incorporated into polypeptides during sperm capacitation, a process that is entirely inhibited by mitochondrial translation inhibitors, but not by cytoplasmic translation inhibitors. They further reported that, unlike 80S cytoplasmic ribosomes, 55S mitochondrial ribosomes are present in polysomal fractions and are actively involved in protein translation in sperm. Furthermore, inhibition of protein translation could lead to significant reduction in sperm motility, capacitation and in vitro fertilization rate. Therefore, contrary to the accepted dogma, nuclear genes are expressed in sperm while in the female reproductive tract until fertilization [35].

Small RNAs are a class of short non-coding RNAs (approximately 19–23 nucleotides) including miRNAs [29]. MiRNAs can regulate gene expression and participate in the regulation of biological processes, such as development, cell proliferation and differentiation, apoptosis and metabolism [36–38], via inhibition/suppression of translation or degradationof mRNA [39]. Severe dysregulation in expression patterns of miRNAs has been observed in different types of reproduction abnormalities [30, 40, 41].

For many decades, the understanding of capacitation was limited to macroscopic observation and description. By and large, the process of sperm capacitation and its underlying molecular mechanisms are poorly understood and require further elucidation [42]. In this study, as the first to utilize next generation sequencing for the study of sperm capacitation,we identified and reported differentially expressed mRNA and miRNA profiles in fresh and capacitated boar sperm. Deep sequencing information was obtained to explore the interaction of miRNA and mRNA and to further understand the underlying mechanism of sperm capacitation.

## Results

### Evaluation of sperm quality parameters

Semen quality parameters were divided into two fractions: FS and CS. Statistically significant difference was detected in FS and CS after induction of sperm capacitation in vitro, as well as insperm acrosome statuses (Table 1). Additionally, the capacitated sperms showed higher motility, viability and acrosome reaction rate compared to fresh sperms (*P* < 0.01).

**Table 1:** Sperm quality parameters of fresh and capacitated boar sperm

Group	Concentration (10^8^ mL^− 1^)	Viability (%)	Motility (%)	Acrosome Reaction (%)
FS	1.932 ± 0.3760	82 ± 0.0274	84.73 ± 0.0328	4.65 ± 0.4848
CS	1.932 ± 0.3760	90 ± 0.0365**	94.03 ± 0.0068**	58.70 ± 0.4686**

Note: **indicates statistical significance at *P* < 0.01. FS, fresh sperm and CS, Capacitated sperm

### Analysis of RNA sequencing

After performing transcriptome sequencing quality control, we obtained a total of 53,686,904 and 59,851,746 raw reads and 26,843,452 and 29,925,873 clean reads in fresh and capacitated sperm, respectively. The uniquely mapped reads to reference genome in fresh and capacitated sperm were 28,565,403 (53.21%) and 30,691,568 (51.28%), respectively. Additionally, in small RNA sequencing, we obtained 18,956,444 and 16,209,736 raw reads, 12,561,033 and 11,222,990 clean reads, and 3,027,230 and 2,944,033 mapped reads to reference genome in fresh and capacitated sperm, respectively. In total, Mirdeep2 detected 1092 unique miRNAs in fresh and capacitated sperm. From these, 259 and 238 were known miRNAs, 769 and 782 were new (novel) candidate miRNAs in fresh and capacitated sperm, respectively (Table 2).

**Table 2:** Overview of transcriptome and small RNA sequencing in fresh and capacitated boar sperm

	Data type	FS	CS
mRNA	Clean reads(pair-end)	26,843,452	29,925,873
	Clean bases	6,642,110,360	7,481,508,592
	% ≥ Q30	86.90	86.06
	Total Reads	53,686,904	59,851,746
	Mapped Reads(single-end)	30,016,749(55.91%)	33,084,783(55.28%)
	Uniq Mapped Reads	28,565,403(53.21%)	30,691,568(51.28%)
miRNA	Raw reads	18,956,444	16,209,736
	Clean reads	12,561,033	11,222,990
	% ≥ Q30	86.43	87.25
	Mapped Reads	3,027,230	2,944,033
	Total-miRNAs	1028	1020
	Known-miRNAs	259	238
	novel-miRNAs	769	782

Note: Clean reads (pair-end), the total number of pair-end reads in clean reads. Clean bases, the total number of bases in clean reads. ≥Q30, the percentage of the base that the quality value of clean data is greater than or equal to 30.Mapped reads (single-end), the number of bases for compared to the reference genome’ clean reads and the percentage in clean reads. Uniq Mapped Reads, the number of reads that are the only place in the reference genome and the percentage in clean reads. Known-miRNA, the number of miRNA is known. Novel-miRNAs, the number of new miRNA for predicted. Total-miRNAs, the number of total miRNA

### GO and KEGG analysis of mRNAs and miRNAs

**Fig. 1:**
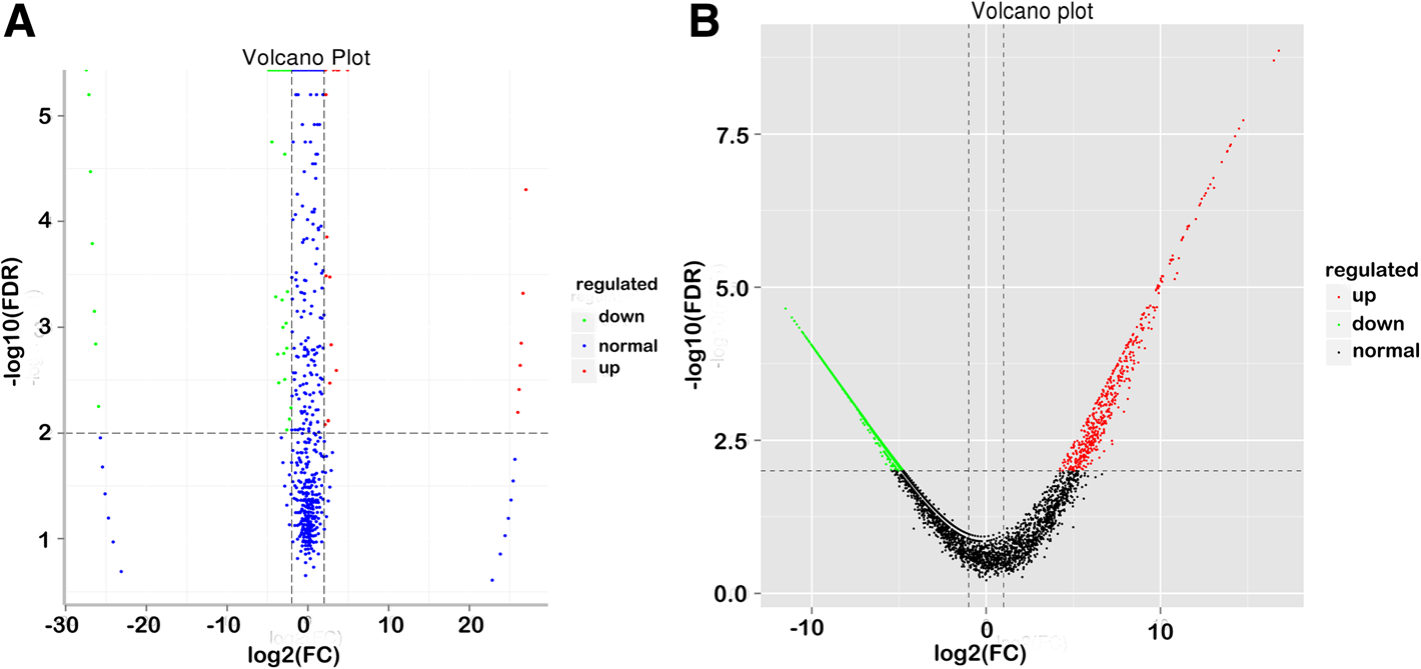
Volcano plot of differentially expressed miRNAs and mRNAs between fresh and capacitated boar sperm. (**a**) miRNAs; (**b**) mRNAs. Each point in the volcanic figure (Volcano plot) represents a gene, numerical value of genes expression in FS and CS as the abscissa, and the negative logarithm of *P*-value-FDR as the ordinate. The red and green dots represent up-regulated and down-regulated differentially expressed genes, respectively. Blue or black dots represent genes that were not differentially expressed

**Fig. 2:**
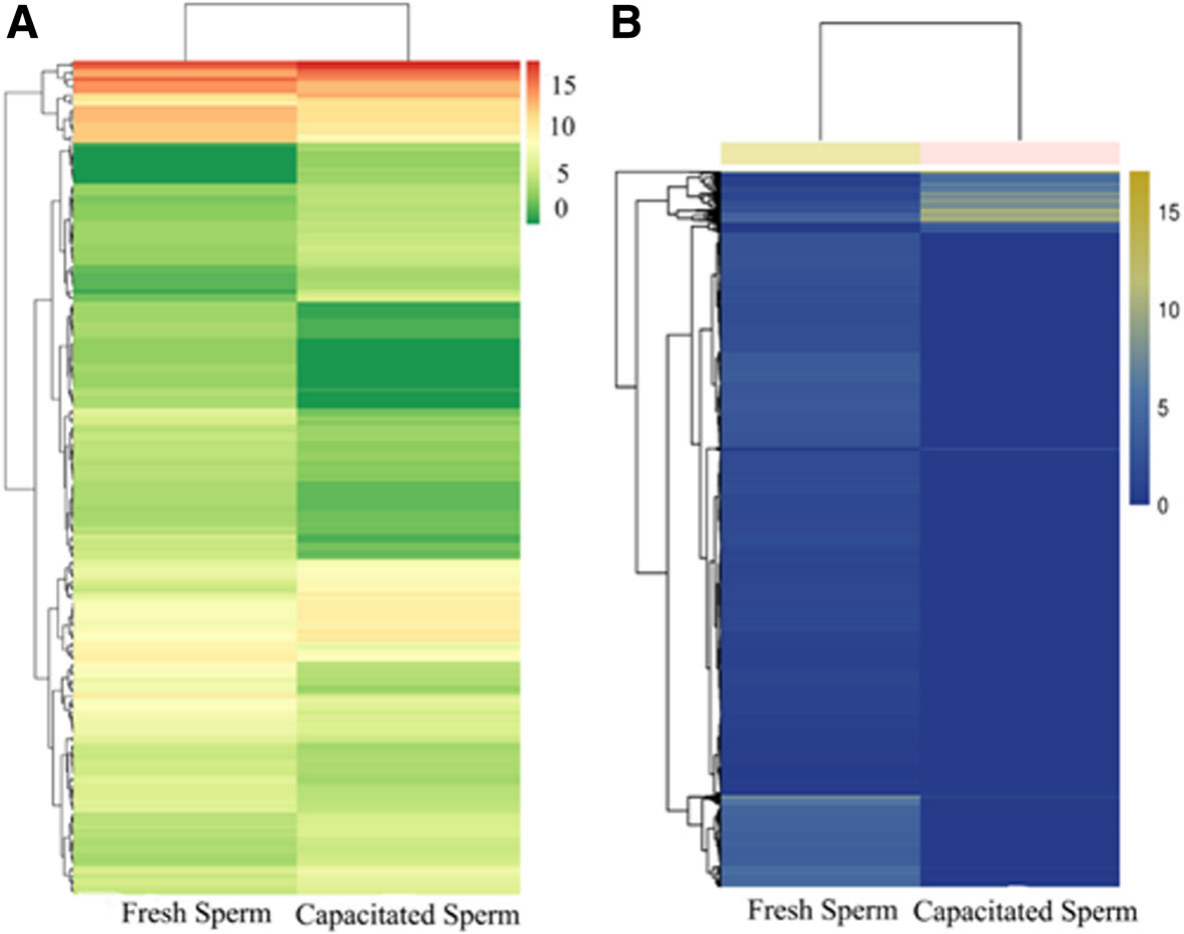
Hierarchical cluster analysis of significantly differentially expressed miRNAs and mRNA in fresh and capacitated boar sperm. (**a**) miRNAs; (**b**) mRNAs. The color represents the level of gene expression, log2 (FPKM+ 1). Chartreuse and blue color denotes high and low expression of genes, respectively. Axes x- and y- represent Euclidean distances and Pearson’s correlation

Log2 (Fold change, expressed in base-2 log ratio of transcript abundance – intensity, ‘log2’) > 1 and FDR (−log10) < 0.01 were selected as standard. Figure 1a and b depict the differential expression between FS and CS. In brief, we identified 5342 differentially expressed mRNAs. From these, 3716 and 1626 were known and novel mRNAs, respectively. Furthermore, 503 mRNAs were upregulated and 4839 were downregulated in FS and CS (Fig. 1b). Results of clustered differentially expressed genes are shown in Fig. 2b. In brief, 69 and 4554 mRNAs were specifically expressed in capacitated and fresh sperm, respectively (Additional file 1: Table S3–1**,** 2, 3).

**Fig. 3:**
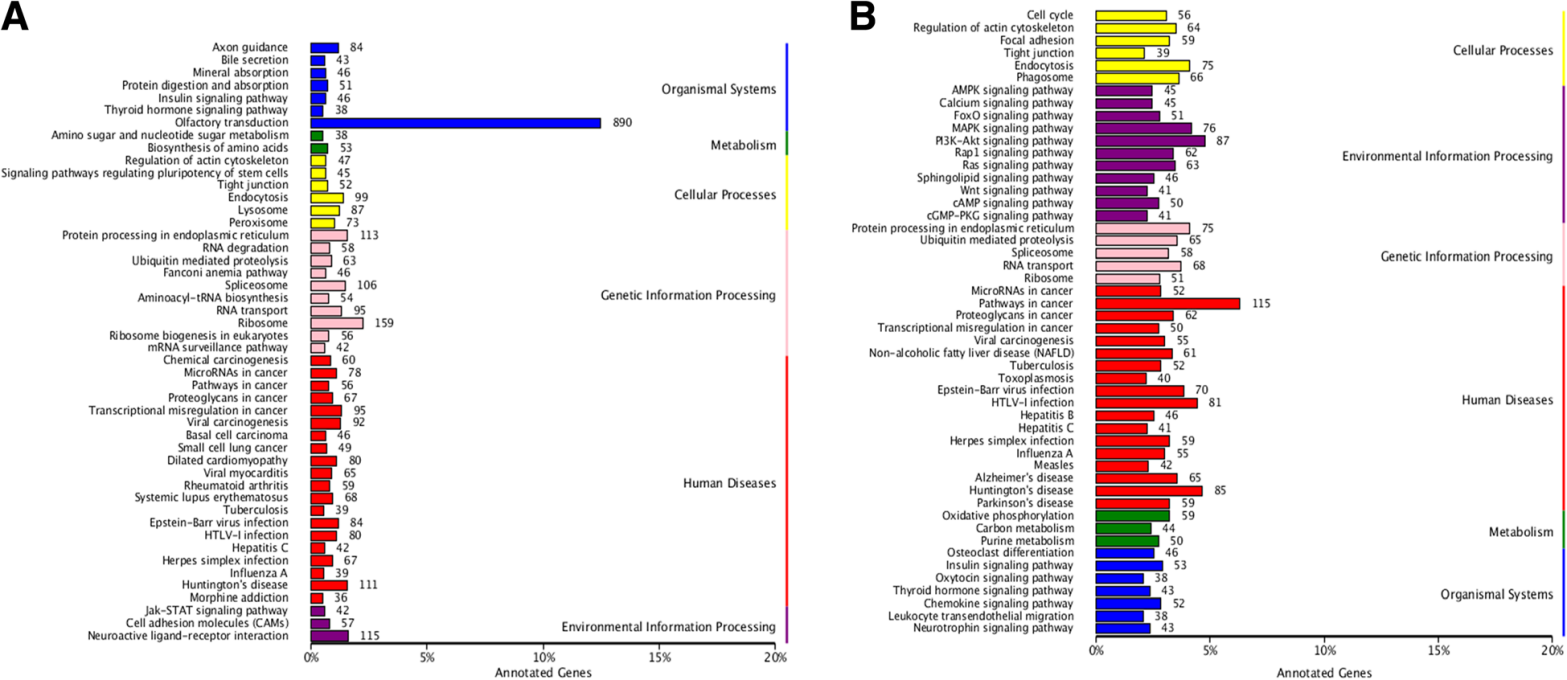
KEGG annotation of differentially expressed target genes of miRNAs and mRNAs. (**a**) miRNAs; (**b**) mRNAs. The vertical axis indicates the name of metabolic pathway of KEGG, and horizontal axis represents the number of genes annotated to the pathway, accounting for the proportion of the total number of genes

Moreover, 5342 differentially expressed genes (DEGs) were analyzed by KEGG. These mRNAs were enriched in 283 pathways, and each enriched pathway contained numbers of differentially expressed mRNAs ranging from 1 to 115 (Fig. 2). From these, 41 mRNAs were enriched in Wnt, MAPK, PI3K-Akt signaling pathways, and energy metabolism and sperm capacitation-related pathways such as Ca^2+^ and cAMP signaling pathways (Fig. 3b).

**Fig. 4:**
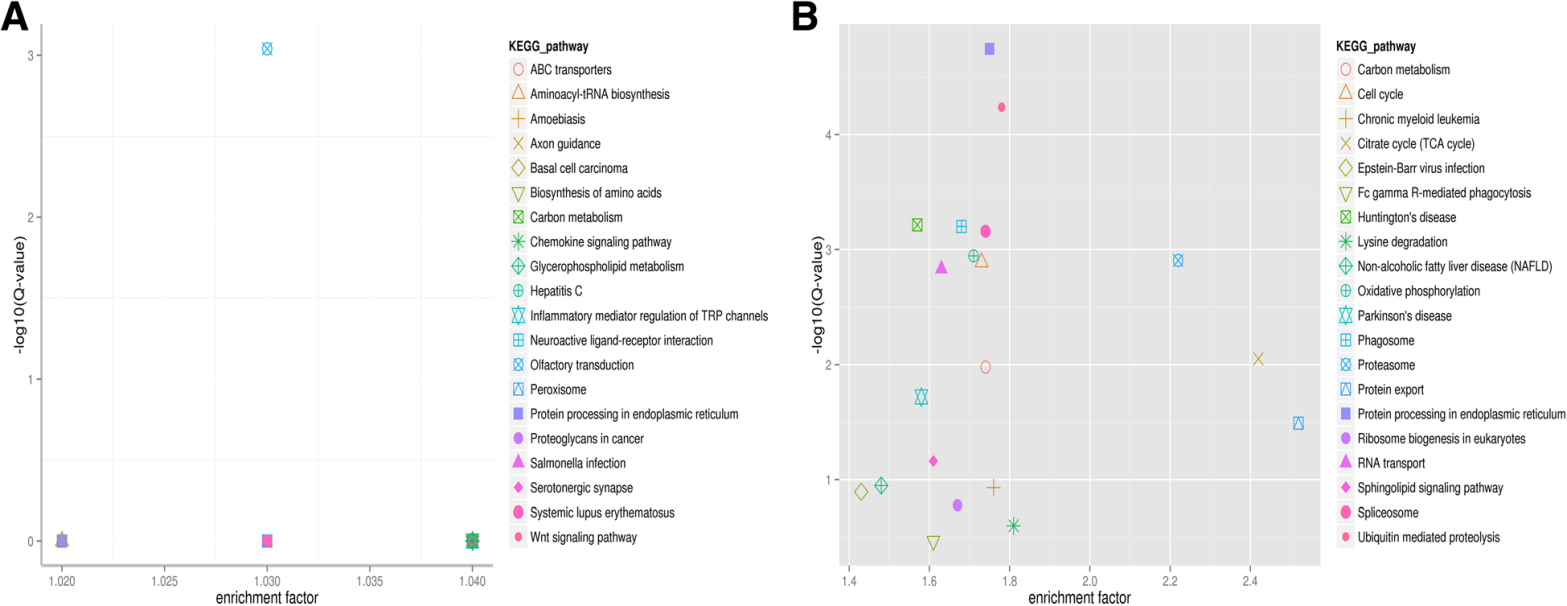
Enrichment analysis of KEGG pathway in differentially expressed target genes of miRNAs and mRNAs. (**a**) miRNAs; (**b**) mRNAs. Each graph in the figure represents a KEGG path, and the accession name is shown in the illustration on the right. The abscissa represents enrichment factor and the ordinate represents Log10 (Q, value)

The enrichment analysis of KEGG pathway in differentially expressed mRNAs was depicted in Fig. 4b. We identified a total of 204 differentially expressed miRNAs (DEM) between FS and CS. Among these, 60 and 141 were known and novel miRNAs, respectively. Whereas 86 and 118 miRNAs were upregulated and downregulated, respectively (Fig. 1a). Hierarchical cluster analysis of differentially expressed miRNAs in fresh and capacitated sperm was shown in Fig. 2a. We further observed that 9 miRNAs (*miR-148a-3p, miR-151-3p, miR-425-5p, miR-132, miR-451, miR7136-5p, miR-489, miR-1343, miR-1306-3p*) and 49 miRNAs exhibited higher expression in CS and FS, respectively (Additional file 1: Table S4). Furthermore, 5 miRNAs (*miR-378b-3p, miR493-5p, miR-133a-3p, miR-362, and miR-214*) were uniquely expressed in fresh sperm.

### Protein internetwork of differentially expressed genes

**Fig. 5:**
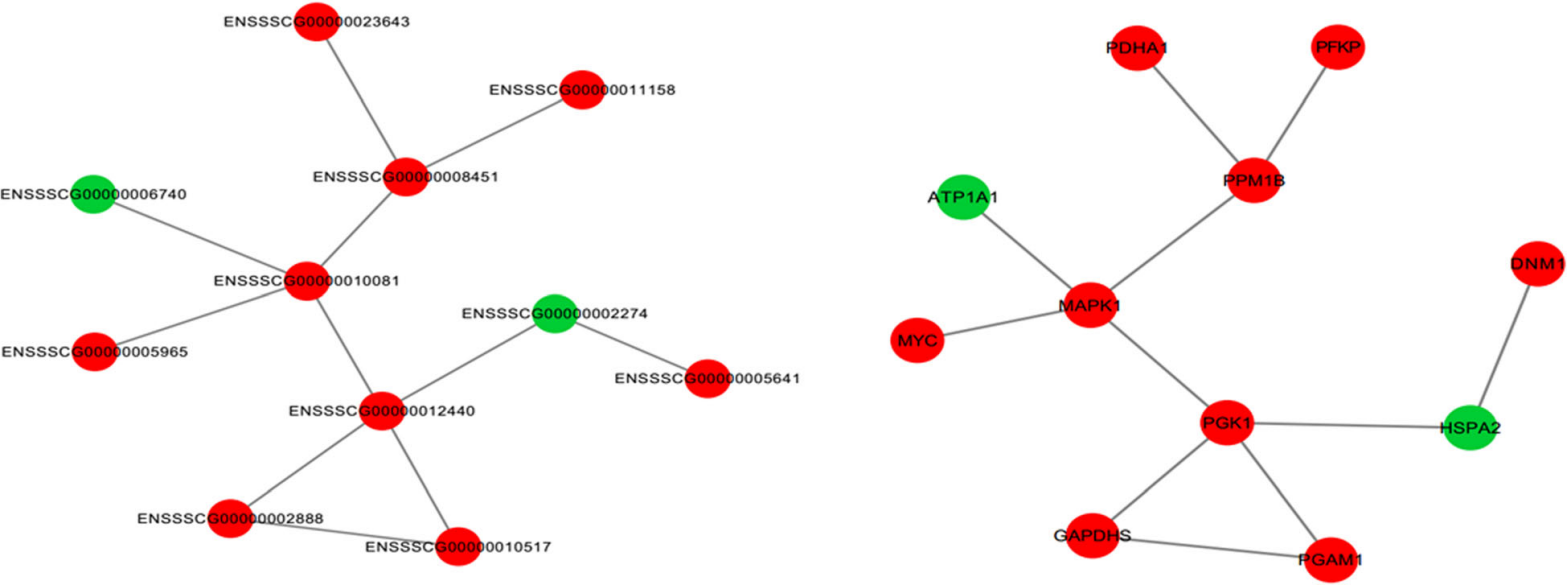
Protein interaction network analysis for the differentially expressed genes between fresh and capacitated sperm. The circle represents the node (differentially expressed protein), the line represents the edge, the red node represents the high aggregation coefficient, and the green node represents the low aggregation coefficient

Based on the protein interaction network, we observed a distinct interactive relationship between the differentially expressed genes. The nodes, degrees, aggregation coefficients and edges in the interaction networks reflect the strength of the interactions between differentially expressed genes. Based on these parameters, we speculated that differentially expressed genes, such as *MAPK1, PGK1, PPM1B,* and *PGAM1,* may play an important role in the regulation of fresh and capacitated boar sperm (Fig. 5).

### Target mRNA prediction and pathway analysis of DE miRNAs

**Fig. 6:**
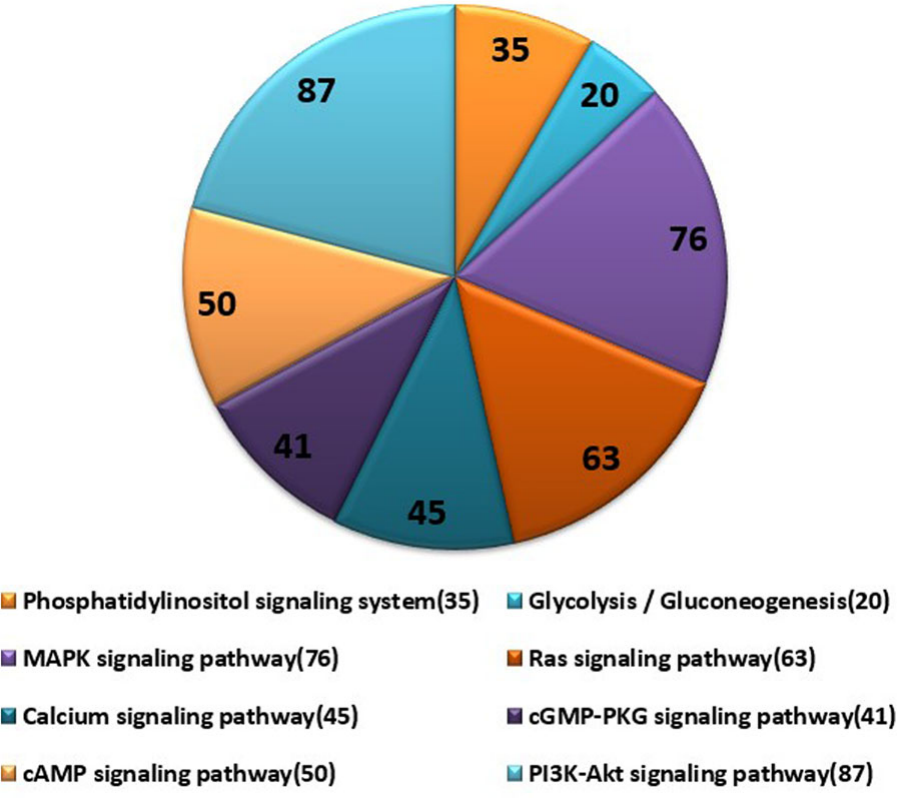
The pie chart of annotated target genes associated with energy metabolism and sperm capacitation signaling pathways. The number represents the number of target genes, and the annotations on the right represent different signaling pathways

In total, we predicted 19,788 target mRNAs using the miRnada and RNAhybrid tools. Canonical pathway analysis further revealed that these genes are annotated to 276 signaling pathways (Fig. 3b). Similarly, these predicted target mRNAs are annotated and associated with energy metabolism and sperm capacitation signaling pathways, including phosphatidylinositol-signaling system, glycolysis, MAPK, calcium, and PI3K-Akt signaling pathway (Fig. 6).

### **qRT-PCR** validation

**Fig. 7:**
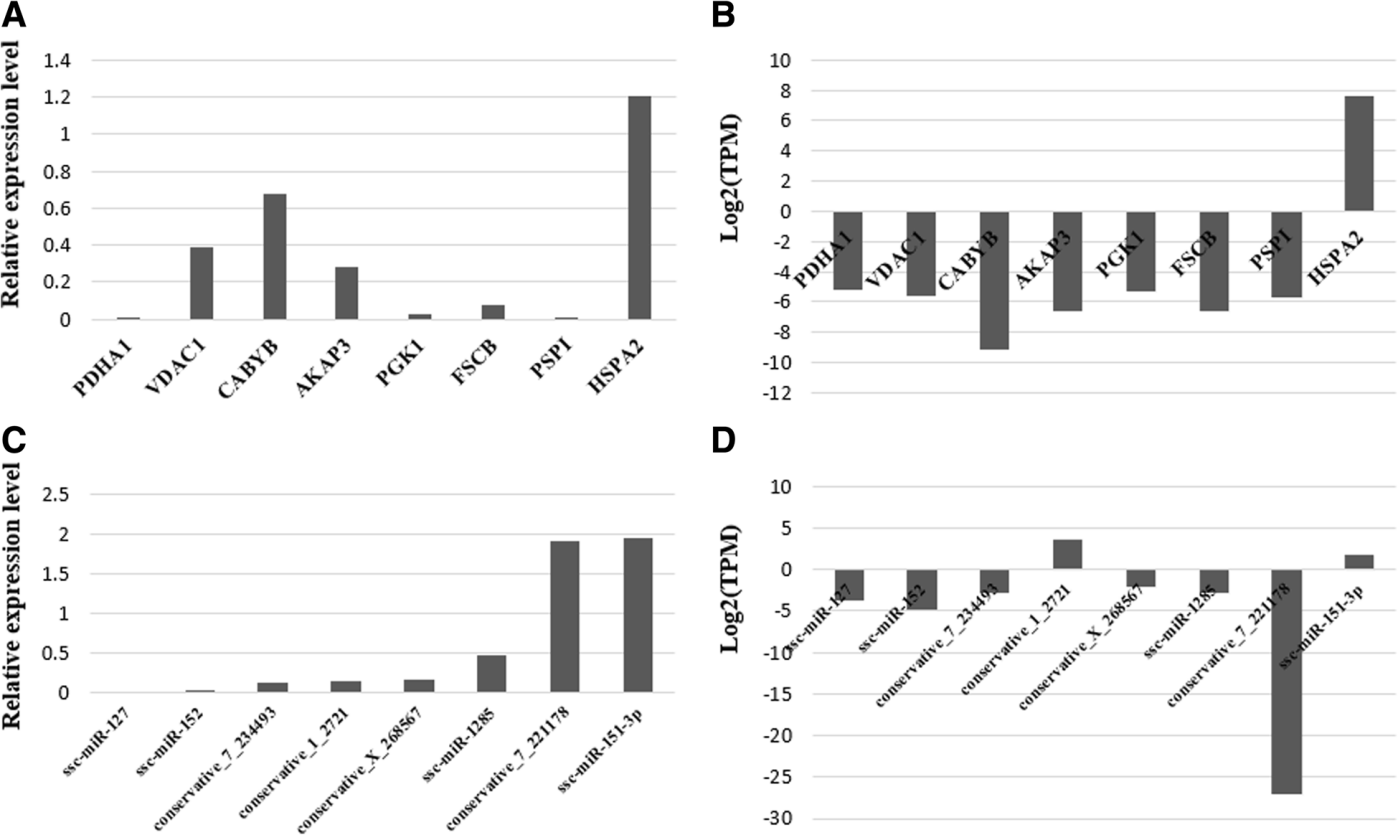
qRT-PCR validation of differentially expressed miRNAs and mRNAs. (**a**) and (**c**): qRT-PCR results of eight differentially expressed mRNAs and miRNAs between fresh and capacitated boar sperm; (**b**) and (**d**): The small RNA and transcriptome sequencing results of eight differentially expressed mRNAs and miRNAs between fresh and capacitated boar sperm

The identified miRNAs and mRNAs (*n =* 8 each) were randomly selected for verifying their expression level in fresh and capacitated sperm via qRT-PCR. The results showed that, except for *conservative-1-2721* and *conservative-7-221178*, the expression levels of all mRNAs and miRNAs in fresh and capacitated sperm are consistent with the results of high-throughput sequencing (Fig. 7c, d).

## Discussion

In recent past, a number of studies have demonstrated that sperm RNAs contribute to spermatogenesis, sperm movement, capacitation, fertilization, and early embryogenesis [34]. The commonly shared characteristics of miRNAs and mRNAs in mammals indicate their important roles in regulation, control and guidance of sperm functions. Building on extensive scruitiny of scientific literature, our study reported, for the very first time, the comprehensive and systemic investigation of the miRNA and mRNA profiles in fresh and capacitated boar sperm using high throughput sequencing. In current investigation, we identified a total of 204 DE miRNAs and 5342 DE mRNAs between fresh and capacitated boar sperm.

Substantial past evidences have indicated that multiple physiological and biochemical changes are involved in the process of capacitation, such as protein tyrosine phosphorylation [4], sperm membrane cholesterol efflux [5], and increase in intracellular pH [8], Ca^2+^ and bicarbonate (HCO3^−^) concentration [9]. Generally, Ca^2+^ and HCO3^−^ are considered as two important biological elements required for sperm capacitation and are believed to promote protein tyrosine phosphorylation. In this study, heparin induces boar sperm capacitation and affects the expression of miRNAs and their target mRNAs. Some of these miRNAs and mRNAs contribute to protein tyrosine phosphorylation and are mainly involved in membrane-related activities such as G-protein coupled receptor activity, signal transducer activity, transmembrane signaling receptor activity in mitochondria.

In the present study, we analyzed the KEGG pathway of DE miRNAs and their target mRNAs. The results showed that these DE miRNAs and their target mRNAs were mainly enriched in the PI3K-Akt, MAPK, cAMP-PKA, and calcium signaling pathways, which are thought to be important for protein tyrosine phosphorylation and sperm capacitation. It has been reported that PI3K-Akt signaling pathway plays an important role in cell cycle growth, development, apoptosis and cancer [43]. In sperms, the actin polymerization and depolymerization processes can mimic sperm capacitation and acrosome reaction [21]. MAPK signaling pathway is reportedly involved in physiological processes, such as cell proliferation, differentiation, variation and apoptosis, and plays an important role in regulating sperm flagellar activity, hyperactivation and acrosome reaction, especially via the ERK (Ras/Raf/MEK/ERK) signaling pathway [22]. Furthermore, activated ion channels, such as Ca^2+^ channel (CatSper), can trigger signal transduction factors that are generally required for intiating the cAMP-PKA signaling pathway and subsequent steps in sperm capacitation [44].

In our study, *miR-1343* was upregulated in capacitated sperm compared to fresh sperm. The target mRNAs of *miR-1343*,*COL11A1* and *PDE4A*, can participate in PI3K-Akt and cAMP-PKA signaling pathways. *AKAP3*, as a target of *miR-1285*, can combine with *PKA* and *PDE4A* to function as skeletal protein in sperm and regulates the concentration of local cAMP and sperm capacitation [45]. *VDAC1* and *HSPA2* are targets of miR-127. They are involved in calcium signaling pathway and MAPK signaling pathway. It has been reported that *VDAC1* is mainly located in sperm mitochondrial membrane and in outer dense fibers of sperm flagella, which affects sperm motility, survival rate, acrosome reaction, capacitation, tyrosine phosphorylation, fertilization and embryo development [46, 47].

Furthermore, some newly identified miRNAs and their targets may be associated with sperm capacitation. *CATSPER4*, the target of *miR-151-3p*, is a sperm-specific calcium channel. CatSper controls the concentration of intracellular calcium and forward movement of sperm [48]. The CatSper channel has been identified in human [49], murine [50], and equine sperm [51]. Recently, it has also been identified in ovine sperm [52]. *CABYR*, a target of novel miRNA (*unconservative_7_234335*), is a calcium binding tyrosine phosphorylated chemical fiber sheath protein involved in sperm capacitation [28]. The binding protein *ACRBP*, a target of new miRNA (*unconservative_11_42222*), promotes the maturation and tyrosine phosphorylation of acrosinthat are closely related to sperm capacitation [53]. One previous study has also demonstrated that the bovine sperm capacitation process requires *AKAP3*-degradation; and the degree of such was regulated by the level of *AKAP3* tyrosine phosphorylation [54]. In our investigation, we found that a number of mRNAs related to sperm capacitation in other species were also differentially expressed in boar sperm. Some important target mRNAs and proteins of miRNAs associated with tyrosine phosphorylation during in vitro capacitationare listed in Table 3. Nevertheless, despite the novel and fascinating findings of our current study, new scientific questions, such as how these DE miRNAs and mRNAs interact with each other to regulate sperm capacitation, remains unanswered and warrants further investigations.

**Table 3:** The miRNAs and their targets involved in signaling pathways and process of capacitation in boar sperm

miRNA	log2FC	Target gene	log2FC	Signaling pathway	Function
miR-1343	1.6303	PDE4A	–	cAMP-PKA	Regulating the concentration of local cAMP and sperm capacitation [45]
miR-1285	−2.9161	AKAP3	−6.5979	MAPK	Regulating the level of AKAP3 tyrosine phosphorylation [71]
miR-127	−3.8252	VDAC1	−5.5646	Calcium	Affected the function of sperm motility, survival rate, acrosome reaction, capacitation, tyrosine phosphorylation, fertilization and embryo development [46, 47]
miR-151-3p	1.7770	CATSPER4	−5.7323	Calcium	Affect the concentration of intracellular calcium and forward movement of sperm [72]
miR-133a-3p	−26.7016	PRDX5	−8.2880	–	Preventing oxidative stress during human sperm capacitation [73]
miR-378	−2.3458	DNM1	−5.0628	–	Regulation of human sperm acrosomal exocytosis [74]
miR-1306-3p	1.5420	CLU	−7.2109	–	Important for sperm maturation and capacitation [75]
miR-214	−25.9240	CYP19A1	−5.4823	–	Increasing the translational activities during capacitation for more protein synthesis [76]
conservative_3_158937	1.0748	MYC	−6.9168		
conservative_7_221178	3.5235	PSPI	−5.7360	–	Participating in the regulation of reproductive immunity, and the process of fertilization; maintaining sperm viability, exercise ability, and mitochondrial activity [77]
unconservative_7_234335	1.7749	CABYR	−9.1633	–	Calcium binding tyrosine phosphoric acid fiber vitamin sheathing protein, participates in sperm capacitation [28]
unconservative_11_42222	1.8879	ACRBP	−4.8677	–	Promotion of acrosin maturation and sperm capacitation [53]

## Conclusion

To conclude, our study is first to provide evidence that in vitro capacitation induces comprehensive changes in expression patterns of miRNAs and mRNAs in boar sperm. We also generated valuable data on novel boar miRNAs and genomic clusters expressed in fresh and capacitated boar sperm. Our findings, along with existing evidences, support the notion that differentially expressed miRNAs and their target mRNAs in fresh and capacitated boar sperm play considerable role in the regulation of sperm apoptosis, mitochondrial membrane potential and spermatogenesis alteration. These discoveries indicate a functional redundancy of these genes in controlling sperm capacitation and thereby, fertility. Our findings provide important insights for the understanding of the RNA profile in boar sperm and future elucidation of the underlying molecular mechanism relevant to mammalian sperm capacitation.

## Methods

### Sperm collection and induction of sperm capacitation

Fresh ejaculates were harvested from 11 sexually mature Landrace boars using manual collection method as previously described [55]. Collection of samples was performed in accordance with the regulations for the Administration of Affairs Concerning Experimental Animals (Ministry of Science and Technology, China, revised in June 2004) and was approved by the Institutional Animal Care and Use Committee in the College of Animal Science and Technology, Sichuan Agricultural University, Sichuan, China, under permit No. DKYB20081003 [56]. The sperm quality parameters were determined with SQA-V (MES, Israel). Only fresh ejaculates with sperm motility greater than 0.8, normal morphology, and sperm concentrations higher than 1 × 10^8^ mL^− 1^ were used in this study. In order to eliminate individual differences in our analysis, five sample pools (*n =* 5) were generated by mixing the fresh ejaculates 2–3 boars for each pool. Then, these ejaculate pools were equally divided into two aliquots. One aliquot (Fresh sperm, FS) was immediately frozen in liquid nitrogen and stored at − 80 °C, and another aliquot (Capacitated sperm, CS) was immediately prepared for capacitation.

Before sperm capacitation, fresh sperms were washed thrice with BTS solution and centrifugated at 600 g for 5 min. Then, fresh sperms were incubated in Tris-buffered medium (TBM, 113.1 mM NaCl, 3 mM KCl, 7.5 mM CaCl_2_·2H_2_O, 5 mM Sodium pyruvate, 11 mM Glucose, 1 mM Caffine, 20 mM Tris, 1 mg mL^− 1^ BSA, and with a final pH 7.6~ 7.8) supplemented with 10 mM heparin at 38.5 °C, 5% CO_2_, 100% humidified incubator for 30 min [57]. Finally, capacitated sperms were immediately frozen in liquid nitrogen and stored at − 80 °C until RNA extraction.

### Sperm viability and acrosome reaction assay

Sperm viability was assessed according to Kovacs and Foote [58] with some modifications. Briefly, 100 μL fresh and capacitated sperms were mixed with isovolumetric and pre-incubated Trypan blue (0.4% in distilled water) and placed in incubator at 37 °C for 2 min. Then, 20 μL of mixture was placed on a glass slide with a cover slip to observe and calculate the percentage of non-colored sperm (at least 500 sperms) under an optical microscope (400×). The capacitated sperms were evaluated based on the changes in pattern of 0.05% Coomassie brilliant blue (CBB) according to the method described by Zhuo et al. with some modifications [59]. Finally, 20 μL-capacitated sperms were smeared on glass slide, air-dried and then soaked in pre-incubated CBB and placed in an incubator at 37 °C for 5 min, then washed with distilled water three times and air-dried. The percentage of capacitated sperms (acrosome reaction rate) was observed (at least 200 sperm) using a phase contrast microscope (1000×) [60].

### Total RNA extraction, library preparation and sequencing

For each sample (*n =* 5), total RNA extraction of fresh and capacitated sperms was performed with Trizol LS Reagent (Ambion, USA) [61]. Briefly, the straws were thawed by plunging into a 37~ 38 °C waterbath for 1 min. The sperms in three straws were collected in a 1.5 mL tube. The sperm suspension was centrifuged at 3400 g and 4 °C for 5 min. The pellets were resuspended with 1 mL of hypotonic solution with 0.5% of Triton X-100 (Roche, Germany). The samples were incubated for 10 min on ice for lysis of the somatic cells. After centrifugation at 5000 g for 5 min, the hypotonic/triton X-100 solution was discarded. Then, 0.75 mL of TRIzol LS reagent was added. The sperm pellets were washed three times with RNase-free PBS, and then resuspended in 0.25 mL of RNase-free water. The concentration and quality of total RNA were measured using NanoDrop ND1000 spectrophotometer (NanoDrop Technologies, USA). The purity (OD 260/280 ≥ 1.8; OD260/230 ≥ 1.0) and concentration (≥250 ng μL^− 1^) of total RNA were qualified for library preparation. Then, small RNA libraries were generated using small RNA Sample Kit Prekit (NEB, USA) according to manufacturer’s instructions. The quality and yield after sample (*n =* 5) preparation were measured with Agilent 2100 Tape Station and Qubit 2.0, and libraries were sequenced on Illumina Hiseq 2500 platform.

### Quality analysis and mapping

Clean data (clean reads) were obtained by removing reads containing adapter, reads containing ploy-A/T/C/G and low qualities reads from raw data. All the downstream analyses were performed on high quality clean data.

The sequence alignment and subsequent analysis using a reference genome were performed using the designated reference genome of *Sus scrofa* (ftp://ftp.ensembl.org/pub/release-75/fasta/sus_scrofa/). Then, clean reads of small RNA were mapped to the *Sus scrofa* genome sequence with miRDeep2 [62]. Clean reads of transcriptome were compared with reference genome by TopHat2 [63]. Information of the location in reference genome or gene, as well as peculiarity of sequence characteristics of the sequenced samples were also obtained.

### MiRNA identification and differential expression analysis

Bowtie [64] software was used to compare clean reads with Silva database, GtRNAdb database, Rfam database and Repbase. Filtered non-coding RNA, including ribosome RNA (rRNA), transport RNA (tRNA), small nuclear RNA (snRNA), small nucleolar RNA (snoRNA), and repeat sequences, and miRNAs of unannotated reads were obtained. Then, Mirdeep2 [62] was used to identify the known miRNA and novel miRNA, and to predict the function of miRNAs. Differential expression of miRNA and mRNA between the FS and CS fractions were analyzed with TPM and FPKM algorithm [65] using the DESeq R package (1.10.1). miRNA and mRNA with an adjusted *P* < 0.01 and absolute value of log2 (Fold change) > 1 were assigned as differentially expressed.

### MiRNA target prediction

For prediction of the potential targets of differentially expressed miRNAs, miRnada [66] and RNAhybrid [67] were used to compare the gene sequence information of corresponding species to the known miRNA and novel miRNA identified in boar sperm.

### GO and KEGG enrichment analyses

GO enrichment analysis was performed on target genes of miRNAs and differentially expressed mRNA usingthe GOseqR package [68]. In addition, the differentially expressed protein coding genes were also analyzed using GO. The enrichment of miRNA target genes and differentially expressed protein-coding genes in KEGG pathways were analyzed by the KOBAS [67] software.

### Quantitative reverse-transcription polymerase chain reaction (qRT-PCR) validation

In order to verify the accuracy of highthroughput sequencing results, we randomly selected and confirmed the expression of 8 miRNAs and 8 mRNAs in fresh and capacitated sperms by qRT-PCR. Fresh and capacitated sperm samples were prepared for resampling by mixing sperms from 2 to 3 individual boars. All primers were either designed based on homologous counterparts in the GenBank database using Primer Premier 5.0 software or adopted from previous literatures (Table 4). U6 [69] and PPIA [70] were used as reference genes, respectively. qRT-PCR was performed using SYBR PrimeScript miRNA RT-PCR Kit (Takara Biotech, China) on a StepOnePlus real-time PCR system (Applied BioSystems, USA) according to our laboratory protocol [56].

**Table 4:** Primers information of miRNAs and mRNAs for qRT-PCR validation

Gene ID	Primer sequences (5′-3′)	Amplicon (bp)	GenBank/miRBase accession
PPIA	F:CACAAACGGTTCCCAGTTTT	174	NM_214353
	R:TGTCCACAGTCAGCAATGGT		
PDHA1	F:GATGATGCAGACTGTTCGCC	138	XM_003360244
	R:TCCGTAGGGTTTATGCCAGC		
VDAC1	F:TGATGGGACGGAGTTTGGTG	115	NM_213960
	R:GGCTGCTATTCCAAAGCGTG		
CABYB	F:AAGTAGCTCACGGTCCTTCG	202	NM_001256771
	R:GGCATACTTGTTGCCACATCC		
AKAP3	F:GCACCCAACAAAAGCCTGAG	96	XM_021090980
	R:GCCGGGAGTCTTATCCGAAG		
PGK1	F:GCTGGACGTGAAGGGAAAGA	104	NM_001099932
	R:CTGACTTGGCTCCGTTGTCT		
FSCB	F:GCTATTGATGAAGCAGCCCC	74	XM_001924913
	R:AGTGAGTGTCTCCTTGGTGG		
PSPI	F:TGGGCCTTGCTGTTCAGT	202	NM_213837
	R:TCCCACAGGTGAGGTTGAGA		
HSPA2	F:TGAGCGGTACAAGTCGGAAG	119	XP_003356782
	R:TCTTGCCCCTCAGTTTCTCG		
ssc-miR-127	TCGGATCCGTCTGAGCTTGGCT	–	MI0013144
ssc-miR-1285	CTGGGCAACATAGCGAGACCCCGT	–	MI0013164
ssc-miR-151-3p	CTAGACTGAAGCTCCTTGAGGA	–	MIMAT0013883
ssc-miR-152	TCAGTGCATGACAGAACTTGG	–	MI0013104
conservative_1_2721	ATTTGTGCTTGGCTCTGTCA	–	Novel
conservative_7_221,178	GGGGGGTGTAGCTCAGTGGTAGAGC	–	Novel
conservative_7_234493	GCTGGGTGCTGGCTGGGGC	–	Novel
conservative_X_268567	TGGCGGGCGGCGGGCGGCGGGC	–	Novel
U6	F:TTATGGGTCCTAGCCTGAC	–	EU520423
	R:CACTATTGCGGGTCTGC		

### Statistical analysis

All results are shown as adjusted least squares means ± standard error means (LSM ± SEM). The mean cycle threshold (Ct) value was converted to relative expression level using the 2^−△△Ct^ method [61]. Statistical analyses of the expression levels of the miRNA and mRNA were performed using unpaired *t* test (SPSS software version 18.0, IBM). Differences of *P* < 0.05 were considered as statistically significant, and differences of *P* < 0.01 were considered as highly statistically significant.

## Additional file

Additional file 1: Raw and collated data. **Table S1.** Differential expression of mRNAs between fresh and capacitated boar sperm. **Table S2.** Differential expression of miRNAs between fresh and capacitated boar sperm. **Table S3–1.** 69 specifically expressed mRNAs in capacitated boar sperm. **Table S3–2.** 4554 specifically expressed mRNAs in fresh boar sperm. **Table S3–3.** 434 co-expressed mRNAs in fresh and capacitated boar sperm. **Table S4.** 49 miRNAs with higher expression level in boar fresh sperm compared to capacitated sperm. (XLSX 818 kb)

## Abbreviations

DEGs: Differentially expressed genes
DEM: Differentially expressed miRNAs
GO: Gene ontology
KEGG: Kyoto Encyclopedia of Genes and Genomes
PTP: Protein tyrosine phosphorylation
qRT-PCR: Quantitative reverse-transcription polymerase chain reaction
ROS: Reactive oxygen species
RPKM: Reads per kilobases of exon region per million mapped reads
